# Spiking neural network model of free-energy-based reinforcement learning

**DOI:** 10.1186/1471-2202-12-S1-P244

**Published:** 2011-07-18

**Authors:** Takashi Nakano, Makoto Otsuka

**Affiliations:** 1 Okinawa Institute of Science and Technology, Onna, Okinawa 904-0412, Japan

## 

Reinforcement learning is a theoretical framework for learning how to act in an unknown environment through trial and errors. One reinforcement learning framework proposed by Sallans and Hinton [1], which we call free-energy-based reinforcement learning (FERL), possesses many desirable characteristics such as an ability to deal with high-dimensional sensory inputs and goal-directed representation learning, and neurally plausible characteristics such as population coding of action-value and a Hebbian learning rule modulated by reward prediction errors. These characteristics imply that FERL is possibly implemented in the brain. In order to understand the neural implementation of the reinforcement learning and pursue the neural plausibility of FERL, we implemented FERL in a more realistic spiking neural network than binary stochastic neurons.

An FERL framework uses a restricted Boltzmann machine (RBM) as a building block. The RBM is an energy-based statistical model with binary nodes separated in visible and hidden layers. In the RBM, due to its connectivity, the posterior distribution over hidden given visible nodes is statistically decoupled, yielding the simple computation of posterior distribution [2]. An RBM is implemented using a spiking neural network with leaky integrate- and-fire neurons. The network is composed of state, action, and hidden layers. The state and action layers consist of several modules (neuron groups) associated with certain states and actions. All state neurons are unidirectionally connected to all hidden neurons. Action neurons are bidirectionally connected to hidden neurons to reflect the selected action to the hidden activations. The action-values, are approximated by the negative free-energy, can be approximated by the firing of the hidden neurons. All connection weights are updated by a Hebbian learning rule and reward prediction error. The agent takes action based on the activation of action neurons.

Our spiking neural network solved reinforcement learning tasks with both low- and high-dimensional observation. All desirable characteristics in FERL framework were preserved in this extension. In both cases, the negative free-energy shows proper representation of the action-values. The free-energies estimated by the spiking neural network have high correlation with one estimated by the original RBM. Activation patterns of hidden neurons reflect the goal-oriented action-based category after reward-based learning (Figure 1).

**Figure 1:**
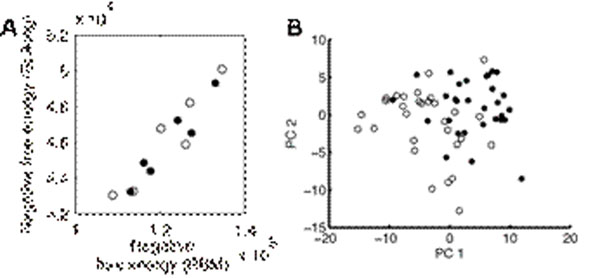
Performance of the spiking neural network. A. The free-energies estimated by both the spiking neural network and the original RBM. They are highly correlated (correlation coefficient, r = 0.9485) B. The hidden neurons activation on the two principal components. The hidden activation patterns are clustered by same optimal action.

## Conclusions

Our spiking neural network implementation of FERL solves reinforcement learning tasks without losing desirable characteristics of FERL. These results suggest the FERL as a candidate of reinforcement learning rule implemented in the brain.
